# Exploring patient experiences with and attitudes towards hypertension at a private hospital in Uganda: a qualitative study

**DOI:** 10.1186/s12939-019-1109-9

**Published:** 2019-12-30

**Authors:** Hayley M. Lynch, Aliza S. Green, Rose Clarke Nanyonga, Darinka D. Gadikota-Klumpers, Allison Squires, Jeremy I. Schwartz, David J. Heller

**Affiliations:** 1 Arnhold Institute for Global Health, Icahn School of Medicine at Mount Sinai, 1216 5th Avenue, New York, NY 10029 USA; 2 Clarke International University, Kampala, Uganda; 3 NYU Rory Meyers College of Nursing, New York, USA; 4 Section of General Internal Medicine, Yale School of Medicine, New Haven, USA

**Keywords:** Hypertension, Non-communicable diseases (NCDs), Uganda, Qualitative research, Health literacy

## Abstract

**Background:**

Hypertension is the leading risk factor for mortality worldwide and is more common in sub-Saharan Africa than any other region. Work to date confirms that a lack of human and material resources for healthcare access contributes to this gap. The ways in which patients’ knowledge and attitudes toward hypertension determine their engagement with and adherence to available care, however, remains unclear.

**Methods:**

We conducted an exploratory, qualitative descriptive study to assess awareness, knowledge, and attitudes towards hypertension and its management at a large private hospital in Kampala. We interviewed 64 participants (29 with hypertension and 34 without, 1 excluded) in English. General thematic analysis using the Integrated Conceptual Health Literacy Model was used to iteratively generate themes and categories.

**Results:**

We identified three main themes: Timing of Hypertension Diagnosis, Aiming for Health Literacy, and the Influence of Knowledge on Behavior. Most participants with hypertension learned of their condition incidentally, speaking to the lack of awareness of hypertension as an asymptomatic condition. Drove nearly all participants to desire more information. However, many struggled to translate knowledge into self-management behaviors due to incomplete information and conflicting desires of participants regarding lifestyle and treatment.

**Conclusions:**

Internal patient factors had a substantial impact on adherence, calling attention to the need for educational interventions. Systemic barriers such as cost still existed even for those with insurance and need to be recognized by treating providers.

## Background

Hypertension (HTN), like other non-communicable diseases (NCDs), is increasingly common in Uganda. A 2018 study reported an age and sex-adjusted nationwide prevalence of 31.5%, and in 2016 the Ministry of Health reported that up to 9% of all hospital deaths in Uganda were attributable to complications of HTN [1, 2]. Current research suggests that the rapid urbanization of Uganda and sub-Saharan Africa (SSA) is largely at fault for the increasing prevalence due to its promotion of more sedentary lifestyles and unhealthy Western diets in the form of fast food chains [3]. Despite its rising prevalence, rates of HTN awareness, treatment, and control remain low [4, 5].

Several studies have attempted to explain this discrepancy through patient-focused qualitative work. Musinguzi et al. conducted interviews with hypertensive patients to this end in the Mukono and Buikwe districts of Uganda. They found that health system access, socioeconomic status, and use of traditional medicines influenced awareness and self-management of HTN. The public and private health facilities serving these patients also struggled with shortages of diagnostic equipment, anti-hypertensive medication and personnel, which served as an added barrier to education and adherence [6]. Another study conducted at a large public referral hospital in Kampala found that participant knowledge of HTN was inversely related to blood pressure control, and in their conclusions similarly attributed poor adherence to a lack of adequate resources [7]. This second study, however, used a binary survey format which limited insight into the subtleties of participant understanding that may also have influenced their behaviors. Studying these nuances and the role of patient understanding in self-management of HTN is important as we look towards interventions, but they are challenging to isolate in resource-limited settings.

Much of the existing research above focuses on public providers, but the private sector is an increasingly important element of healthcare services in Uganda. In 2015, USAID reported that in Eastern, South-Western, and Central Uganda, the majority of Ugandans (53%) visited private facilities for their healthcare needs, as opposed to public facilities (37%) or traditional health practitioners (10%). Research suggests that the main driver of this shift is a sense among Ugandans that the private sector is more readily accessible and provides higher quality care [8]. The government has also recognized the crucial role the private sector plays in expanding healthcare access, and prioritized fostering public-private partnerships [9]. The increasing role of the private sector makes it necessary to understand the factors influencing HTN awareness and control in this healthcare setting.

This study aims to understand the nuances in patients’ experience with, attitudes towards, and knowledge of HTN at a large private hospital in Uganda. These data might provide unique insight into how these factors influence patient behaviors and medication adherence, and may in turn inform the development of interventions aimed at optimizing patient self-management of hypertension across the country.

## Methods

### Study design

We used qualitative description as our study design. A qualitative approach involves interviews with participants, which are uniquely suited to investigate the “why/how/what” of participant behaviors, motives, views and barriers that the research team sought to understand [10]. Qualitative description is a technique within qualitative research used to describe a phenomenon or experience of the participant, wherein the researcher’s interpretation and portrayal strives to stay close to the “surface of the data and events” provided by participants [11]. This provides a wealth of raw data that is particularly useful in assessing needs and designing future interventions [12].

### Theoretical framework

We used the Integrated Health Literacy Conceptual Model to assess the proximal and distal components of health literacy in the Ugandan context and identify links to possible measurable outcomes. Health literacy in this model is understood as a dynamic, multifaceted process that involves the consecutive steps of accessing, understanding, processing and communicating information [13]. Proximal components of interest in our study included participant understanding of HTN, willingness to treat/prevent it, and ability to afford treatment. Distal components included cultural views of HTN, health systems, and access to primary care in this population. These can also be understood as internal and external factors.

### Setting

The study was conducted in a large (110-bed) private, urban hospital in Uganda between June and August 2017. The hospital primarily serves insured upper- and middle-income residents of Kampala and immediate surrounding areas, although not all patients fall into these categories.

### Sample

We sampled patients seen at two of the hospital’s outpatient clinics that treat HTN: a general clinic run by general practitioners (physicians with 1 year of post-graduate internship) and a specialist clinic run by general physicians (physicians with several years’ post-graduate training in internal medicine). We included all patients with self-described proficiency in English and who were at least 18 years old. We included participants both with and without HTN in this study, to gain insight regarding prevention practices and differences in knowledge between the two populations.

### Data Collection & Recruitment

Participants were selected using convenience sampling (i.e., those in clinic that day). We sought to interview 20 to 30 patients with HTN and without, pending theoretical saturation [14]. We recruited facility nurses to introduce the project to participants during vital sign checks to request their participation. If they agreed, researchers [HL, AG] subsequently obtained informed consent and conducted interviews in a private room. Interviews ranged from 10 to 45 min and were audio-recorded and subsequently transcribed.

The interview guide (Additional file 1: Supplemental Material) was developed from the HTN self-management evidence-base by the research team and reviewed by local physicians [15–17]. Conceptually, the instrument covered the core domains of participant self-management by exploring knowledge of HTN, as well as its prevention, treatment and chronic nature. Participants with a diagnosis of HTN were asked additional questions to assess adherence to recommended lifestyle changes and prescribed medications. We iteratively updated the interview instrument based on participants’ responses.

### Ethical review

The study received Institutional Review Board approval from the team’s home institution, the Ugandan study partner, and the country’s IRB.

### Data analysis

We used iterative descriptive and causal coding approaches to guide the analytic process. Descriptive coding assigns a name to a passage that reflects what the participant discusses, while causal coding targets where participants name “causes” or how they attribute their experiences [18]. The latter was important to understand the culturally-driven perspectives of participants about their experiences with HTN. Both processes occurred iteratively. To conduct the coding process, three researchers [HL, AG, DGK] used a sample of selected transcripts to independently generate codes, and then shared these codes with colleagues [DJH, AS, RNC] to generate a standardized codebook. Using this refined codebook, two researchers [HL, DGK] utilized NVIVO software to re-code all interviews and generate reports. We then analyzed these reports for patterns and emerging themes, with final themes and categories generated by team consensus.

## Results

We interviewed 64 participants during the study period. We initially achieved data saturation in patients without HTN at 25 participants. However, new ideas introduced by a concurrent study of providers in the same setting prompted us to amend our interview guide to investigate these new questions and conduct additional interviews in this cohort. Data saturation for these questions was achieved with 9 additional non-HTN participant interviews. To achieve saturation in patients with HTN we altered our methods to recruit only patients with a prior diagnosis of HTN or a high BP reading at the time of visit, which was approved by all IRBs. One participant was excluded after this shift in methodology as he/she was found to not to have a diagnosis of HTN or an elevated BP reading. The final sample size therefore included 29 participants with HTN (14 female, 15 male) and 34 without (15 female, 19 male). Three themes emerged from our analysis: Timing of Hypertension Diagnosis, Aiming for Health Literacy, and the Influence of Knowledge on Behavior.

### Timing of hypertension diagnosis

The timing of when and how participants received their HTN diagnosis emerged as an important theme, as it was largely influenced by distal systemic and cultural factors. Very few participants reported going to the doctor for general check-ups and screening visits, sharing that they did not see the need, it was not part of their culture, or it was not covered by insurance. A few articulated a combination of these factors:

*“Healthcare is very expensive, and coming to a medical physician for just a checkup when you don’t really feel you have any health issue bothering you, it might be perceived as luxury … they will say insurance does not actually accept that, does not allow people to come be checked when they are not actually sick.”* (17–018, non-HTN).

Other participants agreed that a well-checkup is a luxury for the wealthy, rather than a routine part of preventive healthcare.

Perhaps as a result of these factors, only two of the 29 participants with HTN were diagnosed at a general checkup, both required by their work or school. The rest were diagnosed during evaluation for other issues, such as an accident, post-partum care, or another chronic condition. Several participants expressed frustration with the lack of information they were given about the importance of screening for asymptomatic diseases like HTN. When prompted, several reported a desire to have general checkups done.

### Aiming for health literacy

Participants with and without HTN were aware of gaps in their knowledge about the condition and sought out information to close them. Driving this desire for most participants was their view of HTN as a serious condition, citing long-term effects such as stroke and death experienced through friends and family members. Several participants voiced concern about the asymptomatic nature of HTN: “So many have died because of high blood pressure, especially if it strikes and you’re not aware. It strikes at a very high speed, so I know it is one of the most dangerous, which can kill someone” (17–033, non-HTN). The understanding of HTN as a “silent killer” contributed significantly to non-hypertensive participants’ desire to learn more about prevention, curability, and how to help others in case of an emergency. Participants with HTN also frequently asked about how to prevent its lethal effects but were equally concerned by the need for long-term medication. As a result, they often sought out information about whether they could discontinue their medications and if their HTN could be cured.

Sources of knowledge differed between participants with and without HTN. Those with HTN often received most information from their doctor, supplemented by the internet or their community. However, they were frustrated by the lack of information provided by their doctors before their diagnoses: “Nobody would ever sit you down to explain to you that high blood pressure is this and this, like the way they do about HIV. Nobody does until you are a victim” (17–060, HTN). Participants without HTN echoed this sentiment, with most reporting that they had found all of their information from the internet, media, or through acquaintances with HTN. There was a general concern for the lack of awareness in the greater community: “If at all I had known how to control, or to avoid it, I believe some of my people wouldn’t have gotten pressure. I would have tried all means to avoid and maybe to educate them” (17–032, non-HTN). Participants specifically called on the hospital, media, and government to take on the role of education as they had done with infectious diseases.

### The influence of knowledge on behavior

Participants translated their knowledge into behaviors, but multiple internal and external factors impacted this process. These interactions were most apparent with respect to lifestyle changes and medication adherence.

#### Lifestyle changes

Participants with and without HTN reported an understanding of how diet and exercise influence blood pressure, but often had trouble implementing these changes. With respect to diet, participants with HTN often provided detailed descriptions of the foods they should avoid: “I have to avoid alcohols, I have to take very little salts … I try to avoid even fatty meat” (17–014 HTN). Non-hypertensive participants were more likely to describe broader categories of “fatty” or “oily” foods to avoid. However, participants in both groups reported struggling to follow these recommendations when they ate food provided by work or at restaurants that they did not prepare themselves. They also struggled to avoid eating unhealthy foods that they liked.

Many also expressed knowledge of the importance of exercise, but participants with and without HTN both found this to be the most difficult lifestyle change to implement, primarily due to lack of time. Some participants addressed this issue by integrating exercise into their lives in creative ways, such as gardening or walking to work instead of driving. Aside from time constraints, several cited frustrations with the perceived failure of exercise and diet alone to control their blood pressure: “I was actually exercising. That’s why I can’t really pinpoint why it persisted. Because I was exercising and then my diet, it’s more organic than junk really” (17,047, HTN). Their lack of results, in turn, contributed to lack of adherence to these lifestyle changes.

Many participants cited stress as a common cause of HTN. Unlike perceptions of dietary or exercise changes, they often described stress as something that was impossible to prevent: “In the society which is affected by poverty, one has no way of survival … The person is always miserable and as I told you earlier, one of the causes of high blood pressure is too much worries” (17–043 non-HTN). A few participants commented on this kind of societal stress (i.e. poverty), while others cited individual stressors such as trouble at work or an ill family member. All forms and examples of stress reported, however, were viewed as inevitable.

#### Medication adherence

Unlike lifestyle changes – which participants recognized as important but were unable to act upon – poor medication adherence was often secondary to limitations in understanding of its role in managing HTN. More than one third of participants with HTN reported extended periods of time where they did not take their medications at all, most often in the first few months after diagnosis. Participants commonly cited unawareness of the chronic nature of the disease and need for lifelong treatment due to lack of symptoms as the reason for this pattern. One participant understood only after her third doctor’s visit that she was expected to take medications indefinitely:

*I was advised to take the first medication the first month … I don’t think I was told to come back for review. I was just given medication for a month, then I just walked away. The next time I came, I think I had so many complications … she took my pressure that time I think it was 170 over something, it was high, it was very high. So, I was given some amlodipine, so after it was finished I never came back for review, because I didn’t know I was supposed to. So, the next time I came, I think it was still high. She told me, ‘You know what? I think you have to be taking this for life.’* (17–057, HTN).

Others similarly reported reaching this understanding only after multiple visits with physicians where their blood pressure was found to be high, or after experiencing complications from the disease. Two participants experienced miscarriages as a result of this misunderstanding.

However, even those who had been followed by a doctor for years regarding their HTN and reported good adherence did not seem to have a complete grasp of the fact that medications served to control rather than cure high blood pressure. As one hypertensive patient asked, “What [is the] doctor’s advice? Taking drugs all the time and exercising the body, which I practice and I do … Sometimes I come and they tell me [the blood pressure] is okay, why don’t I leave this drug?” (17–020, HTN). Others reported insidious patterns of non-adherence, where they would decide to go on and off of the medication themselves based on their pressure readings. They were often then concerned by the fact that the blood pressure would rise whenever they were off medications: “Sometimes I can be ok for some months without taking medication but afterwards the pressure rises again. That worries me” (17–062, HTN). This same participant had previously stated that they understood the need for long-term medication, demonstrating an apparent discrepancy in his/her understanding and how it translated to behavior. That participant explained, “With time I accepted that’s the way I need to keep my life … that’s how I started taking the medication” (17–062, HTN). Similar discrepancies were apparent in other interviews, and were only made evident upon probing into participant adherence patterns.

These discrepancies were often linked to an underlying desire in participants to stop taking medications, often fueled by concerns about their long-term effects on the body: “When you’re constantly taking them, I imagine it has got to have some negative impact on the body somehow. Because these are chemicals really … it’s not food” (17–063, HTN). The effects of medications appeared to be as distressing to participants as the long-term effects of uncontrolled HTN.

Certain factors appeared to increase participant understanding of the need for long-term medication, which often resulted in improved adherence. Those most commonly cited were 1) a lack of improvement in their blood pressure at a subsequent visit; 2) explicitly hearing someone tell them to stay on medication for life; 3) a desire to avoid symptoms and complications. Financial barriers, however, impeded medication adherence independent of patient understanding. The cost of lifelong treatment and follow-up care – which arose from individual circumstances as well as broader systemic factors – often precluded patients from being adherent. While most had insurance to cover their medications, several participants cited periods of non-adherence due to inability to pay for their drugs. Many noted that a lifelong, daily medication is particularly costly. As one participant reported: “Yeah it’s a lot of money. Because if you are to take it every day for the rest of your life … and if you have another 5 years to live, that’s a lot of money” (17–051, HTN). Others added that other fiscal considerations sometimes prevented them from purchasing medications: “There are times you have other demands … This is a drug which you are supposed to have almost every day but now imagine the time when because of certain things, you don’t have the money. At times it’s a challenge” (17–030, HTN). Even participants with insurance at the time of interview did not always have a stable source of funding for HTN treatment, contributing to stress and medication non-adherence.

Participants also reported structural issues that increased the cost of care, primarily the large amount of time away from work required to attend follow-up appointments. Long wait-times to see the physician meant many participants had to take full days off from work to come to one appointment: “Time is money. So, you have told me one hour, okay I’ll wait. But then I waited from 10 up to 4. It was abnormal.” (HTN, 17–050). The substantial investment of time required to follow-up with the doctor and renew prescriptions was often a critical extrinsic barrier to medication adherence.

## Discussion

The Integrated Model of Health Literacy offers a useful framework for examining NCD care, as patients with chronic diseases must rely heavily on their own knowledge as well as their abilities to navigate the healthcare system to get the care they need. This study identified multiple proximal and distal components of participant health literacy and reveals the nature of how they relate to behaviors and adherence patterns.

Prior studies in Uganda have attributed poor blood pressure control to factors distal to the patient, such as lack of availability of medications, follow-up care, and insurance [19]. However, our data demonstrates that nuances in participant understanding and the desire to stop taking medications also contribute significantly to insidious patterns of non-adherence, even in motivated patients. These proximal components of health literacy may be tackled through patient education initiatives. The nearly unanimous desire for more knowledge suggests that educational interventions – both for those with and without HTN – would likely be well-received.

Education early in disease course specifically may prevent misunderstandings regarding the chronic nature of HTN and promote medication adherence, as many participants identified periods of non-adherence in this period. This goal may prove difficult, however, in a system where most patients are diagnosed with HTN incidentally while seeing the doctor for other complaints. Increased use of wellness checkups may address this issue by creating dedicated time for providers to educate patients about chronic conditions. This move would require a cultural shift regarding the nature of doctor visits, as well as a strategy to cover the costs.

A potential intervention that addresses multiple needs identified in this study is the implementation of nurse-led Medication Adherence Clubs, or MACs. MACs were first utilized in the treatment of HIV to encourage open discussion and reduce clinical congestion at clinics so more patients could be seen without increasing the number of physicians [20]. They also provide a dedicated time for disease-specific education. There is now early evidence of MACs increasing adherence and follow-up amongst patients with NCDs in East Africa, suggesting this model as a promising, cost-effective approach for our study setting [21, 22].

However, a major limitation of this approach is the voluntary nature of participation in these groups. A potential platform to reach beyond this self-selecting population is through local faith-based organizations (FBOs), as studies report that such programs have achieved statistically significant changes in reducing cholesterol and blood pressures of participants [23]. In the context of SSA, studies have demonstrated the efficacy of FBOs in promoting knowledge and utilization of family planning, and others have posited that FBO’s are well-situated to intervene in the realm of preventing cardiovascular disease in this setting [24, 25]. One participant interviewed here mentioned that his/her church was affiliated with hospitals and hosted “health camps,” (17–017, HTN) suggesting that such a strategy could be feasible in this setting.

Future research should replicate this study in other public and private facilities in Uganda, to determine if themes are persistent across contexts and with different patient groups demographically. Nonetheless, the current study can inform intervention design specific to the study’s site and contribute to the foundational literature for developing interventions at the community level - as well as informing public health strategies to address the growing hypertension crisis in the country.

### Limitations

This study was conducted at a large, private urban hospital, where most patients are insured: a setting not easily generalizable to most other parts of Uganda. However, this allowed us to delve further into patient-specific factors of understanding and non-adherence unhindered by external forces. Participants were often well-educated, with consistent access to the internet – which is not the case for many Ugandans. We also captured only patients who came into the hospital, who may have a greater understanding of and motivation for HTN treatment than those who missed appointments. These patients though still had misunderstandings about HTN and HTN management and wanted to learn more, exhibiting a need for disease education likely widely applicable throughout Uganda. Additionally, lack of translation service limited our interviews to English-speaking participants. However, almost all patients at this hospital were fluent English-speakers. That the interviews were conducted by non-Ugandan researchers may have influenced participant responses, particularly regarding concern about HTN and adherence. The coding process was also carried out primarily by non-Ugandan members of the research team, presenting a potential for bias in the analysis given the team’s pre-existing beliefs about barriers to HTN care and the importance of general wellness checkups.

## Conclusions

Interviews with Ugandan private hospital clients revealed many barriers to HTN control, even among those able to access and afford consistent outpatient care. Patients often learn about their disease incidentally; receive incomplete or misleading information about how to control it; and face internal struggles regarding whether and how to apply the knowledge they acquire. These challenges influence their behavior in turn, especially regarding medication adherence. Education prior to and at the point of diagnosis is critical to address these misunderstandings, thereby promoting prevention and adherence. Our analysis demonstrated that this population would be eager to learn and amenable to educational interventions. Systemic factors such as the costs of care – both financial and through lost time and wages – also limit adherence to HTN treatment. Due to persistent poor or unstable insurance coverage in Uganda, this barrier may require more public-private partnerships to develop as part of a solution as private facilities are poorly equipped to address it alone. Providers should also aim to be aware of how these barriers impact their patients when providing care and making recommendations.

## Additional file

**Additional file 1.** Supplemental Material: Patient Interview Guide.

## Data Availability

The de-identified interview transcripts with or without coding generated during this study are available upon request made to the corresponding author.

## Abbreviations

FBO: Faith-Based Organization
HTN: Hypertension; or to label a participant as a person with hypertension
MAC: Medication Adherence Clubs
NCD: Non-Communicable Disease(s)
Non-HTN: A participant without hypertension
SSA: sub-Saharan Africa
