# Supplementary material for: Exploring patient experiences with and attitudes towards hypertension at a private hospital in Uganda: a qualitative study
Source: Int J Equity Health. 2019 Dec 30;18:206. doi: 10.1186/s12939-019-1109-9 (PMC6937689; doi:10.1186/s12939-019-1109-9)
Supplement: Supplementary file 1 — Additional file 1. Supplemental Material: Patient Interview Guide. [file 12939_2019_1109_MOESM1_ESM.docx]

1. Have you ever received a diagnosis of high blood pressure?

***If they answer yes:***

1. What have you been told about your high blood pressure?
2. What do you think high blood pressure means? (probe: complications, where does condition come from?)
3. Are you concerned about your high blood pressure? (probe: complications)
4. Do you think you will always have high blood pressure?
5. Do you take medication for high blood pressure?
6. Which one?
7. How often?
8. Where do you get it?
9. How much does it cost?
10. Are there ever times you do not take your medication? Why? (Probe: difficulty of getting medication, feeling healthy/not in pain, running out…etc).
11. Are you aware of any connection between diet and high blood pressure?
12. Are you aware of any connection between exercise and high blood pressure?
13. Has a doctor ever talked to you about diet and exercise as part of your treatment plan?
14. Have your habits changed at all since you were told you had high blood pressure? (probe: diet, exercise, meds, doctor visits)
15. How often are you coming to appointments here?
16. How often are related to high blood pressure condition?
17. If you do not come, what prevents you from coming?
18. Is it easy to get here?
19. Do you find your care is affordable?
20. Is there anything that you think can be done to improve care of your high blood pressure?

*If extra time: questions about referral process*

1. When and where were you first told you had high blood pressure? Who told you?
2. How did you end up being treated for high blood pressure at IHK (probe for: referral process).

***If they answer no:***

2. Do you know anyone with high blood pressure?

a) If yes, what do you understand about their diagnosis?

1. Do you view high blood pressure as a serious condition?
2. Are you concerned about getting high blood pressure?
3. Are you aware of any connection between diet and high blood pressure?
4. Are you aware of any connection between exercise and high blood pressure?
5. Has a physician here ever counseled you about prevention of high blood pressure?
6. If yes, what did they say?
7. If yes, have your habits changed at all since that discussion? Why/why not?
8. Is it easy to get here?
9. Do you find that your care is affordable?
10. How often are you coming to appointments here?
11. If you do not come, what prevents you from coming?
12. Is there anything that you think can be done to improve care of your high blood pressure?
